# Quadricuspid aortic valve diagnosed by multidetector computed tomography (MDCT)

**DOI:** 10.1259/bjrcr.20150040

**Published:** 2015-07-16

**Authors:** S K Bellamkonda, A Pasumarthy, S Velicheti

**Affiliations:** Department of Radiodiagnosis, Dr. Pinnamaneni Siddhartha Institute of Medical Sciences and Research Foundation, Krishna District, Andhra Pradesh, India

## Abstract

A quadricuspid aortic valve is very rare form of congenital cardiac valvular disease with an incidence between 0.003 and 0.043% and often incidentally found during echocardiography, surgery or on post-mortem examination (Feldman BJ, Khandheria BK, Warnes CA, Seward JB, Taylor CL, Tajik AJ. Incidence, description and functional assessment of isolated quadricuspid aortic valves. Am J Cardiol 1990; 65 : 937–8). Its diagnosis is often missed, even with the transthoracic echocardiogram, as in this patient. We report a case of a quadricuspid aortic valve that was incidentally found by 256-slice electrographically-gated multidetector row CT/tomographic angiography during screening for coronary artery disease.

## Clinical presentation

A 47-year-old asymptomatic male patient went to the echocardiography laboratory for routine examination in view of a history of hypertension and diabetes mellitus. The patient had a strong family history of coronary artery disease.

**Figure 1.**
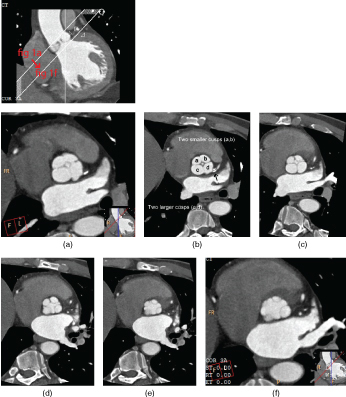
Long axis image of the left ventricular outflow tract showing cross-section images ranging from valve cusp to annulus ring. (a–f) Quadricuspid aortic valve images ranging from valve cusp to annulus ring in diastole showing mild non-coaptation of valve cusps that corresponds with mild aortic regurgitation. Two equal large and small valves are seen. All four valves are functional. (b) Mixed density plaque is noted at the proximal left main stem coronary artery (black arrow).

**Figure 2.**
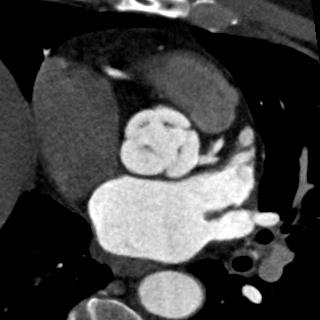
Quadricuspid aortic valve images in systole.

On transthoracic echocardiographic examination, mild aortic regurgitation was identified. Subsequently, a 256 slice multidetector row CT angiogram with calcium score determination was performed. Mixed density partially calcified plaques were noted in the left circumflex artery, left anterior descending artery and left marginal arteries. A quadricuspid aortic valve with two large and smaller cusps (Hurwitz and Roberts anatomical classification type C) was also demonstrated along with mildly impaired coaptation of the valve cusps (Figures 1 and 2).

Due to the asymptomatic status of the patient and the presence of only mild aortic regurgitation, the patient was treated conservatively with antihypertensive medication and antidiabetic medication. The patient remained under clinical follow-up to monitor for deterioration of aortic regurgitation.

## Discussion

Quadricuspid aortic valve was first identified on autopsy by Balington^1^ in 1862. The aortic valve is formed in the aortic trunk by the inward growth of three pads of mesenchymal tissue to form semi-lunar cusps. Abnormal cusp formation results from either aberrant fusion of the aorticopulmonary septum or abnormal mesenchymal proliferation in the common trunk, resulting in abnormal aortic valve cusps.^2^

The clinical significance of a quadricuspid aortic valve arises from its frequently abnormal function. Aortic incompetence appears to be the most prevalent haemodynamic abnormality accounting for 50–75% of the cases, whereas valvular stenosis is rare.^3^ Quadricuspid aortic valves have been reported in association with other cardiac abnormalities, including non-obstructive cardiomyopathy, pulmonary valve stenosis, ventricular septal defect, fibromuscular subaortic stenosis and supravalvular stenosis with left coronary artery atresia.^4^

Hurwitz and Roberts classified a quadricuspid aortic valve into seven different subtypes based on the relative size of each cusp.^5^ While type A and B are considered the most common types of quadricuspid aortic valves,^6^ our patient demonstrated a type C configuration with two equal smaller and larger cusps (Table 1).

**Table 1.** Hurwitz and Roberts’ classification.^1^

Type	Description
A	Four equal cusps
B	Three equal cusps and one smaller cusp
C	Two equal larger and smaller cusps
D	One large, two intermediate and one small cusp
E	Three equal cusps and one larger cusp
F	Two equal larger and two unequal smaller cusps
G	Four unequal cusps

While the majority of patients may be treated conservatively with combined clinical and imaging follow-up, surgical intervention is indicated in symptomatic patients with severe aortic regurgitation or asymptomatic patients with severe left ventricular dysfunction or dilatation.^7^ Surgical options include tricuspidalization or formal aortic valve replacement.

## Learning points

1. Quadricuspid aortic valve closely resembles bicuspid aortic valve/tricuspid aortic valve. Therefore, it is necessary to pay close attention to detect its presence.
2. Quadricuspid aortic valve is associated with other congenital abnormalities, such as anomalies of the coronary ostia, non-obstructive cardiomyopathy, pulmonary valve stenosis and ventricular septal defect.
3. Aortic regurgitation complicates a quadricuspid aortic valve, which may require surgical intervention later in life. Therefore, early identification prevents cardiac dysfunction due to aortic regurgitation.
